# Comparison of the Migration Potential through Microperforated Membranes of CD146+ GMSC Population versus Heterogeneous GMSC Population

**DOI:** 10.1155/2021/5583421

**Published:** 2021-03-11

**Authors:** Mohamed Al Bahrawy

**Affiliations:** ^1^Stony Brook University, NY, USA; ^2^Oral Medicine and Periodontology Department, Faculty of Dentistry-Ain Shams University, Cairo, Egypt

## Abstract

**Background:**

Guided tissue regeneration (GTR) is a powerful modality for periodontal regeneration, but it blocks the periosteum and gingival stem cells (GMSCs), from supporting periodontal wound by the nutrients, growth factors, and regenerative cells. The microperforated membrane considered a rewarding solution for this major drawback; GMSCs can migrate through a GTR microperforated membrane toward a chemoattractant, with the blocking of other unfavorable epithelial cells and fibroblasts. In the absence of a sole marker for MSC, a homogeneous population of GMSC is difficult to isolate; using CD146 as confirmatory markers for MSC identification, testing the behaviour of such homogeneous population in migration dynamics was the question to answer in this study.

**Materials and Methods:**

GMSCs from healthy crown lengthening tissue was isolated (*n* = 3), its stem cell nature was confirmed, CD146 and CD271 markers were confirmatory markers to confirm homogenous stem cell population, and magnetic sorting was used to isolate GMSC with CD146 markers. A homogenous CD146 population was compared to heterogeneous GMSCs of origin; the population doubling time and MTT test of the two populations were compared. Migration dynamics were examined in a transwell migration chamber through 8 *μ*m perforated polycarbonic acid membrane, and 0.4 *μ*m and 3 *μ*m perforated collagen-coated polytetrafluoroethylene membrane (PTFE) and 10% fetal bovine serum (FBS) were the chemoattractants used in the lower compartment to induce cell migration, were incubated in a humidified environment for 24 hours, then migrated the cell in the lower compartment examined by a light and electron microscope.

**Results:**

GMSCs fulfilled all the minimal criteria of stem cells and showed low signal 10% for CD146 on average and extremely low signal 2% for CD271 on average. Magnetic sorting optimized the signal of CD146 marker to 55%. GMSC CD146 population showed nonstatistically significant shorter population doubling time. CD146 homogeneous population migrated cell numbers were statistically significant compared to the heterogeneous population, through 0.4 *μ*m and 3 *μ*m perforated collagen membrane and 8 *μ*m perforated polycarbonate membrane. Scanning electron microscopy proved the migration of the cells.

**Conclusions:**

A subset of the isolated GMSC showed a CD146 marker, which is considered a dependable confirmatory marker for the stem cells. In terms of GMSC migration through the microperforated membrane, a homogeneous CD146 population migrates more statistically significant than a heterogeneous GMSC population.

## 1. Introduction

Periodontitis is a chronic inflammatory bacterial infection, where the oral flora organizes a biofilm subgingivally, which constitutes a continuous challenge to the host immune system that responds by continuous inflammatory cytokine shower that affects the body homeostasis; with time, deregulated immune response eventually results, and a hyperresponsive immune reaction causes the destruction of the tooth-supporting apparatus, leading to tooth loss. Periodontitis is considered an irreversible degenerative disease of the odontogenic supporting tissue; this throws light on the immune-mediated nature of periodontal disease [1].

A historical debate did exist about the periosteum's role in bone growth, repair, and regeneration. Two theories have been contrasting; one postulated that periosteum is an inert membrane covering the bone with no exact role [2]; the other considered the periosteum as a functioning membrane with osteogenic potentials, responsible for regeneration and bone growth. A number of classical experiments created strong evidence that leads to modern literature where the essential role of periosteum for bone healing was understood. A classical study when the periosteum surrounding fractures removed the result was the absence of callus in the fracture [3].

Guided tissue regeneration technique (GTR) was based on blocking the growth of unfavorable cells from invading the periodontal wound, namely, the gingival epithelium and connective tissue, but as collateral damage to this technique, were scalding the alveolar bone from its periosteum by elevating a full-thickness flap. Blocking the periodontal wound by a barrier membrane from the periosteum in fact excludes the wound area from a powerful regenerative source which is an essential source of blood and nutrient supply; besides that, the periosteum is a niche of biologic mediators and progenitor cells essential for the regeneration process [4].

Not only periosteum but GTR also deprive the periodontal wound from the gingival connective tissue, to block the rapidly proliferating fibroblasts which can invade the periodontal wound before the slowly proliferating periodontal ligament and bone cells, but regrettably, a well-recognized population of stem cells named the gingival mesenchymal stem cells (GMSC) is blocked from the wound [5, 6] if GMSCs allowed migrating to the periodontal defect and induced to differentiate; using the suitable biological factors into periodontal ligament cells and osteoblasts with a well-designed organized scaffold, biological factor release cascade, such as a system, would satisfy the real aim of GTR. Thus, the occlusive barrier membrane of the classical GTR is unfavorable for periodontal regeneration [4].

In comparison to bone marrow stem cells, the gold standard, the first stem cell described, and the most studied, GMSC was superior in nearly every aspect, besides its ease of harvesting with very low morbidity; no scaring; homogeneous population; high proliferation rate without the need for special growth factors; morphology stable within successive passages; reduced senility; and stable karyotype, maintains its telomerase activity to later passages, and shows very low tumorigenic potential [5]; this concluded that the gingiva is a very good source of stem cells compared to the bone marrow, with functionally competent MSCs, that can be used with a wide range of medical applications.

Gamal and Iacono's clinical study tested macroperforated GTR and posted improved clinical outcome, followed by a series of studies in 2014 and 2016; they hypothesized that GTR membrane perforation allowed bone morphogenic protein (BMP-2) and platelet-derived growth factor (PDGF-BB), besides vascular endothelial cell growth factor (VEGF) and other nutrients migrating freely through the membrane, which was the reason for the improved clinical outcomes [7–9]. In 2018, Al Bahrawy et al.'s in vitro study concluded that macroperforation jeopardizes the GTR membrane occlusive function and its mechanical properties; this study postulated a new development of Gamal and Iacono's concept by a microperforated membrane, a concept was proved, GMSCs can migrate through the microperforation under chemoattractant influence, and the membrane was occlusive for cell migration in the absence of a chemoattractant; hence, the GTR membrane could be a selective occlusive barrier allowing the migration of the desired cells while blocking others, under the influence of the right chemoattractant [4].

A cell to be considered stem cell must be multipotent, can differentiate to other cell types than the tissue of origin, must be clonogenic, and has strong proliferation power. In fact, only a fraction of the plastic adherent cells showed these characteristics; this is explained by the heterogeneous population of the isolated cells and attributed to the nature of tissue of origin [10]. Another issue to consider is the absence of one specific marker that can identify stem cells from other mature cells; many surface markers, for example, CD73, CD90, CD105, CD146, or even neural crest markers; and intracytoplasmic markers like STRO-1, OCT-4, Nanog, Nestin, and Notch-1 that could be used to characterize stem cells [11, 12], taking into consideration that different stem cells from different tissue origins show a different set of markers, but as a minimal criterion, cells must show a high signal of CD73, CD90, and CD105 together. The main drawback with these three markers was that they are expressed by fibroblast, although in weak signal [13]; besides, fibroblast morphology was identical to MSCs; both did plastic adherence and fibroblast dipotency, can differentiate to at least two other cell lines, and made identification of fibroblast from MSC in vitro not an easy task; this urged the need for at least additional cell marker.

It was well described that MSC is located around blood vessels; in 2008, Covas and colleagues compared MSC from different tissue origins to fibroblasts and pericytes, and they concluded that 12 MSC populations were very similar to 4 fibroblasts and 2 pericyte populations phenotypically; the only difference was fibroblast weak signal of the CD146 surface marker compared to MSC and pericytes; both showed a strong signal of this marker in flow cytometry. Comparing the 3 cells genetically, they concluded that the gene expression pattern of MSC is similar to pericytes and stellate hepatic cells, not fibroblast which showed the gene expression pattern of myofibroblasts and smooth muscle cells [14].

Another evidence of the pericyte and MSC similarity was proved in other studies, where the 3 minimal markers of MSCs CD73, CD90, and CD105 have a vascular and perivascular distribution pattern in vivo [15, 16]; to confirm this assumption, other MSC-specific markers were examined, namely, CD146, NG2, Stro-1, and 3G5, which confirmed the previous results of the vascular and perivascular distributions of these markers [15, 17]. Connecting all of this data together, we can conclude the close nature of MSC and pericytes in vivo in contrast to fibroblast; this data built strong evidence that CD146 which is essentially a pericyte marker would be a good candidate to confirm MSC; besides the other three fundamental markers, a cell population expressing them all with a high signal is a homogeneous MSC population.

From all of what was mentioned, we conclude that depriving the wound area from GMSC with its multipotent abilities was not a good idea because it is an important source of regeneration. It has undenied the breakthrough the GTR technique had achieved in the periodontal treatment in general and in the regeneration concept, in particular, but it is now clear the GTR by its traditional occlusive membrane is not the best practice for the regeneration procedure, and microperforation of this membrane is essential; besides, utilizing a full system of specific chemoattractant in the periodontal wound side of the membrane for GMSCs will let this powerful cells invade the wound area, to achieve the optimum outcome of GTR technique, with organized chronologically activated cell differentiation induction biological factors.

## 2. Materials and Methods

### 2.1. Sample Selection

**Figure 1:**
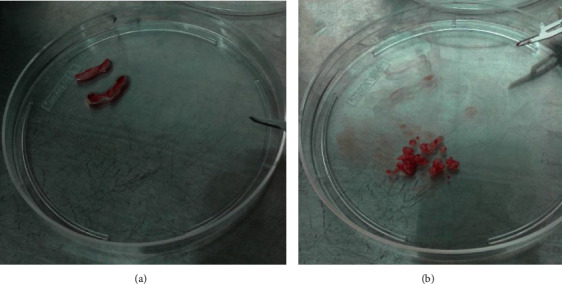
(a) Healthy gingival tissue specimen of discarded crown lengthening procedures. (b) Gingival connective tissue was meshed to 1 mm pieces using a surgical blade.

Gingival specimens are healthy gingival tissue of discarded crown lengthening procedures of outpatients who attended at Stony Brook University dental care clinics Figure 1(a). A parallel case-control experimental study of two groups was designed. Four subjects accepted to participate in this study, all experiments were done in triplicate (*n* = 3), and participants were informed about the nature of the experiment and verbally accepted the use of their discarded tissue in stem cell research. The ethical committee of scientific research at School of Dentistry Ain Shams University and Stony Brook University had approved this study (IRB 575741).

The gingival epithelium was carefully scalded from the specimen; the connective tissue was meshed to very small pieces using the surgical lancet Figure 1(b) then digested in 2 mg/ml Dispase II overnight at 4°C (Sigma-Aldrich, St. Louis, USA) and then in Collagenase IV (Fisher Scientific, Massachusetts, USA) for 40 minutes at 4°C; the resulted cell suspension was strained in 40 *μ*m strain to remove the impurities, then centrifuged at 1200 rpm for 8 minutes. The resultant single-cell suspension was inoculated in 10 cm cell culture dish, in alpha-minimal essential medium (alpha-MEM 1×, Gibco, Thermo Fisher Scientific, Massachusetts, USA), supplemented with 10% fetal bovine serum (FBS) (Hyclone, Thermo Fisher scientific, Massachusetts, USA) and 50 U/ml penicillin G with 50 *μ*g/ml streptomycin and 2.5 *μ*g/ml amphotericin B (Fungizone, Thermo Fisher scientific, Massachusetts, USA), at a concentration of 60 cells/cm^2^.The single-cell suspension plates were incubated in 37°C, 5% CO_2_ humidified incubators. Cells reached confluence after approximately 28 days for the first passage, then subcultured in a P100 plate for the next passages, and reached confluence on average in 14 days.

### 2.2. Colony-Forming Unit

At passage five, cultured cells were detached using 0.05% trypsin/EDTA, cells were diluted in alpha-MEM (Gibco, Thermo Fisher Scientific, Massachusetts, USA) enriched with 10% FBS (Hyclone, Fisher Scientific, Massachusetts, USA) at a concentration of 10^3^ cell/ml in P10 dishes, and media were changed every 3 days and examined under a microscope till typical fibroblast colonies of 100 cells formed.

### 2.3. Population Doubling Assay

Menicanin et al.'s protocol was followed; briefly, GMSCs were seeded in 24-well plate with a concentration of 5 × 10^3^ cells/cm^2^; when 90% confluence was reached, cells were detached using 0.05% trypsin/EDTA and then counted, cells were diluted and reseeded with the same concentration in another 24-well plate, and the same procedure was repeated for five passages. Cells were counted in each passage, and population doubling was calculated using this formula: log^2^ final cell number/log^2^ seeding cell number [18].

### 2.4. Flow Cytometry Assay

At the 5th passage, cell culture was washed twice by PBS, then detached using 0.05% trypsin/EDTA; detached cells were resuspended in 1% bovine serum albumin as a blocking buffer for half an hour. Cells were aliquoted with a concentration of 1 × 10^5^ cells in two test tubes, then 2 *μ*g/ml of CD73 and its isotype control fluorescein isothiocyanate- (FITC-) conjugated mouse monoclonal antibodies in each tube, and then incubated for 30 minutes in 4°C (BD Pharmingen, San Jose, California, United States). The same procedure was done with CD90 and its isotype using APC-conjugated mouse monoclonal antibodies (BD Pharmingen, San Jose, California, United States), for CD105 and its isotype using Alexa 555 gout anti-mouse monoclonal antibodies (Dako, Agilent, Santa Clara, USA) and finally, for CD146 and CD271 and their isotype PE-conjugated mouse monoclonal antibodies(BD Pharmingen, San Jose, California, United States). After incubation buffer was aspirated, cells were washed twice by resuspension in PBS and centrifugation at 1200 rpm for 8 minutes, and cells were then transferred to a flow cytometry facility for the analysis of stem cell marker expression. Regarding the hematopoietic markers, namely, CD14, CD34, and CD45, the same protocol was followed with no difference.

### 2.5. In Vitro Differentiation Assay

#### 2.5.1. Osteogenic Differentiation

**Figure 2:**
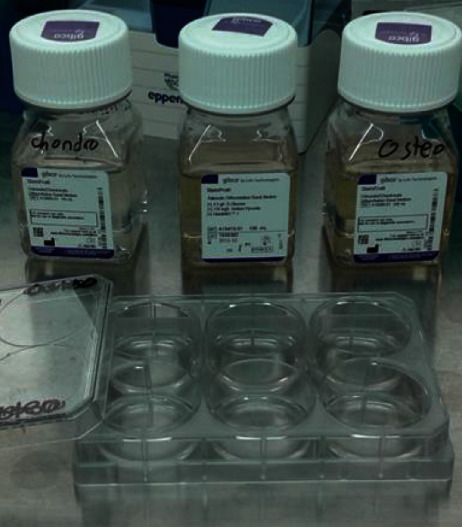
Ready-made osteogenic, adipogenic, and chondrogenic stem cell differentiation media (Gibco StemPro, Thermo Fisher Scientific, Massachusetts, USA).

Cell suspension at passage 4 was seeded in six-well plates with a concentration of 8 × 10^3^ cells/cm^2^ in a ready-made osteogenic induction medium (Gibco StemPro, Thermo Fisher Scientific, Massachusetts, USA), according to Gronthos et al.'s protocol; the medium was changed every 3 days for 28 days in humidified incubators ([19, 20]), the cell cultures were washed twice by PBS, fixed by 4% paraformaldehyde for 1 hour, washed again twice by distilled water, finally stained by 2% Alizarin Red for 45 minutes, finally washed 4 times by distilled water and 2 times by PBS, and checked under a microscope (Figure 2).

#### 2.5.2. Adipogenic Differentiation

Cell suspension at passage 4 was seeded in six-well plates with a concentration of 8 × 10^3^ cells/cm^2^ in a ready-made adipogenic induction medium (Gibco StemPro, Thermo Fisher Scientific, Massachusetts, USA) according to Pittenger et al.'s protocol; the medium was changed 2 times per week for 28 days [21]. After that time, the cell cultures were washed twice with PBS, fixed by 4% paraformaldehyde for 1 hour, and washed again twice by distilled water; the cell culture is washed by 60% isopropanol for 5 minutes, then stained for 5 minutes by Oil Red O in isopropanol (300 mg Oil Red in 100 ml isopropanol), washed by tap water, and finally stained by hematoxylin for 1 minute, again washed by tap water, and checked under a phase-contrast microscope (Figure 2).

#### 2.5.3. Chondrogenic Differentiation

Cell suspension at passage 4 was seeded in six-well plates with a concentration of 8 × 10^3^ cells/cm^2^ in a ready-made chondrogenic induction medium (Gibco StemPro Thermo Fisher Scientific, Massachusetts, USA); the medium was changed 2 times per week for 28 days. After that time, the cell cultures were washed twice by PBS, fixed by 4% paraformaldehyde for 1 hour, washed twice by distilled water, then stained in dark with Alcian blue (10 mg in 60 ml ethanol with 40 ml acetic acid) overnight; the next day, the cell culture was destained by 120 ml ethanol with 80 ml acetic acid for 20 minutes, finally washed 2 times by PBS, and then checked under a microscope (Figure 2).

#### 2.5.4. MTT Assay

Detached cell culture of the fourth passage was suspended in 500 *μ*l alpha-MEM (Gibco, Thermo Fisher Scientific, Massachusetts, USA) enriched with 10% FBS (Hyclone, Fisher Scientific, Massachusetts, USA), poured in spectrophotometer tube, and left in a humidified incubator (37°C, 5% CO_2_); the negative control was a medium-enriched tube without cells, within the same incubator. The next day, 100 *μ*l of MTT stain was added, and tubes were incubated for another 4 hours; then, media were aspirated and 1000 *μ*l of dimethyl sulfoxide (DMSO) was added, and tubes were analysed by a spectrophotometer at a 595 nm wavelength.

### 2.6. Cell Sorting

#### 2.6.1. Flow Cytometry Cell Sorting

The same protocol of cell characterization was followed; the only difference was not to fix the cells; it has to be noted that the sorting procedure was done as soon as possible after cell detachment, as the cells were suspended in serum-free media; finally, after sorting by the machine, cells were collected in media enriched with 20% FBS.

#### 2.6.2. Magnetic Sorting

**Figure 3:**
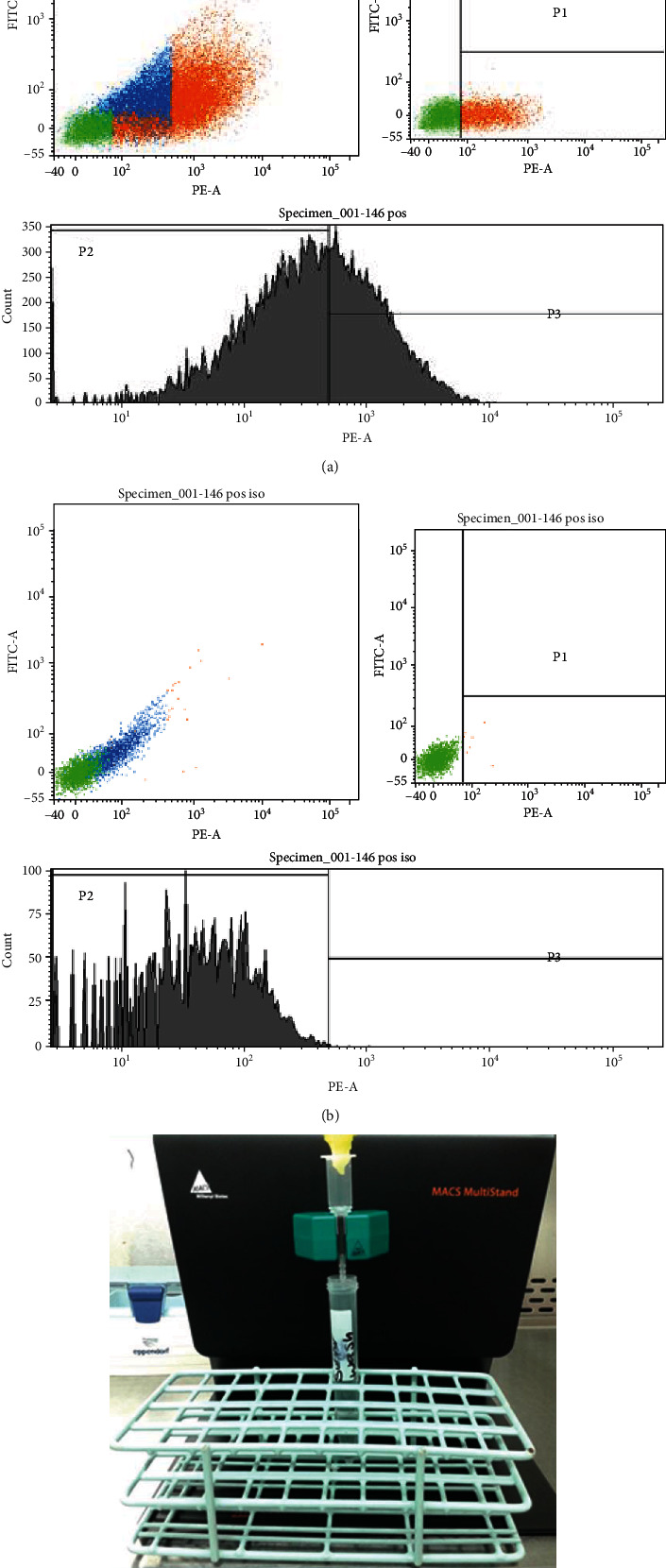
(a) The confirmatory flow cytometry graph of the magnetic sorted GMSC homogeneous CD146-positive population, which optimized the signal to 55% purity. (b) The flow cytometry graphs of the negative control isotype, which showed no signal of the CD146 marker. (c) The magnetic sorting kit.

After detaching, cells were counted and suspended in 1 ml of buffer of the cell sorting kit (MACS cell separation, Miltenyi Biotech, USA). Cells were centrifuged at 300 × g for 10 minutes, the buffer was suctioned, 20 *μ*l Fc block was added, and 20 *μ*l CD146 marker was labelled with microbeads. Tubes were incubated in 4°C for 14 minutes, washed in 1 ml buffer, and centrifuged at 300 × g for 10 minutes. Cells were suspended in 500 *μ*l MAC buffer solution. The magnetic sorting column was primed by 500 *μ*l MAC buffer solution, the cell suspension was added in the sorting column, and the column was washed three times using 500 *μ*l MAC buffer solution in each. Finally, we plunged out the cells from the sorting column using serum-free alpha-MEM Figure 3.

### 2.7. Migration Assay

#### 2.7.1. Microscopic Perforated Membranes

In the transwell chemotaxis migration chamber (Boyden chamber) was the test used to analyse the migration dynamics of GMSCs (Corning Life Sciences, New York, USA);2 types were used, namely, 12 mm perforated collagen-coated polytetrafluoroethylene (PTFE) membrane with a pore size of 0.4 *μ*m and 3 *μ*m pores and a 6.5 mm perforated polycarbonate membrane with a pore size of 8 *μ*m. Cultured heterogeneous GMSCs were the positive control group, and the homogeneous CD146-positive sorted and expanded GMSCs were the experiment group. Cells were detached using 0.05% trypsin/EDTA, then suspended in serum-free alpha-MEM diluted to 1 × 10^4^ concentration and added to the upper compartment of the chemotaxis chamber inserts. For both groups, the lower compartment of the chemotaxis chamber received alpha-MEM with 10% fetal bovine serum as a chemoattractant (Hyclone, Fisher Scientific, Massachusetts, USA), The migration chamber plates were incubated for 24 hours in a humidified atmosphere (37°C, 5% CO_2_).

The next day, the migration inserts were collected from the plates, and media in the upper compartment aspirated. The inserts were washed two times in PBS. Using a cotton swab, the upper side of the membrane was scraped thoroughly to remove all the cells still attached to the upper compartment. Cells on the lower side of the membrane were fixed by 4% paraformaldehyde for 2 minutes; inserts were washed 2 times in PBS; cells were then permeabilized by 100% methanol and stained by crystal violet (1% in 80% ethyl alcohol, Sigma Aldrich) and washed again two times in PBS; membrane was examined under the microscope at 40x magnification; migrated cells were counted in 5 different areas; and the average was counted.

#### 2.7.2. Scanning Electron Microscope

Membranes were cut off the migration inserts, fixed by 4% paraformaldehyde for 2 minutes, and left to dry. Membranes were soaked in 50%, 70%, 80%, 90%, and 100% ethyl alcohol for 10 minutes each, finally frozen in minus 80°C overnight, then sent to an electron microscope facility, were coated by gold, and examined by the electron microscope.

#### 2.7.3. Statistical Evaluation

All statistical analyses were done using SPSS v20 program, IBM; the descriptive analysis was used to determine the distribution of the data, and graphs were plotted; according to it, the Mann–Whitney *U* test was the test of choice according to the data distribution, the alpha significance of difference was set at *p* < 0.05, and all experiments were done in triplicate.

## 3. Results

### 3.1. Colony-Forming Potential

**Figure 4:**
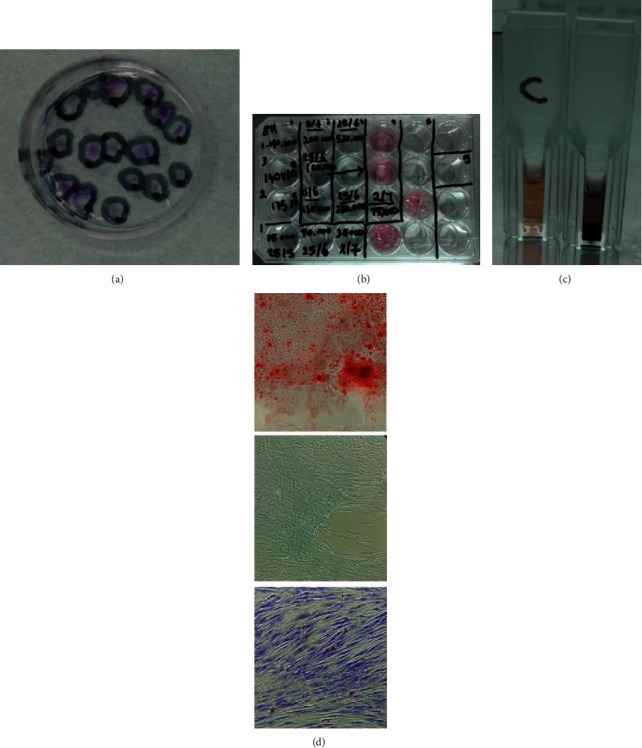
(a) Representative image of CFU experiment showing stem cell colony-forming potential. (b) Representative image showing the cell population doubling potential. (c) Representative image for the MTT essay showing black deposits in the experiment tube compared to the control group. (d) Representative image showing cell differentiation potential; calcium deposition (upper), cartilage glycoprotein deposition (middle), and fat droplet deposition (lower).

Seeded GMSCs in P10 dish showed typical distinctive fibroblast-like colonies of 50 to 100 cells/colony after on average 14 days of culturing in vitro; all experiments were done in triplicate (*n* = 3) for each group; no significant difference was noted between the heterogeneous GMSC group and CD146-positive homogeneous GMSCs in shape, form, or number of cells in colonies (*p* > 0.05; Mann–Whitney *U* test); the only difference noted was that the homogeneous CD146-positive group reached 100 cell colonies 1 day earlier on average compared to the heterogeneous group (Figure 4(a)).

### 3.2. Population Doubling Potential

The two groups were similar in showing strong proliferation capability. The population doubling time was nonsignificantly less in the CD146-positive homogeneous group compared to the heterogeneous group (*p* > 0.05; Mann–Whitney *U* test) (Figure 4(b)).

### 3.3. Cell Characterization

**Figure 5:**
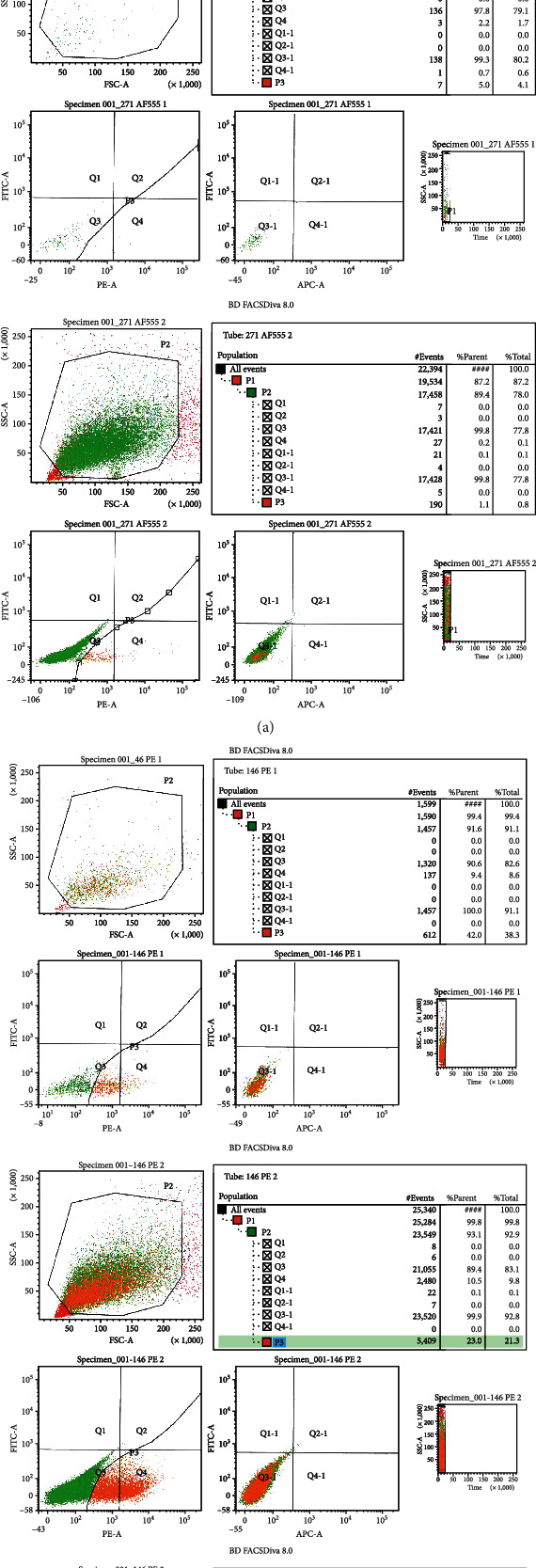
(a) The CD271 flow cytometry graphs of 3 cell lines of the heterogeneous GMSC population, signal percentage of cell line A: 2% (upper), cell line B: 1% (middle), and cell line C: 4% (lower). (b) The CD146 flow cytometry graphs of 3 cell lines of the heterogeneous GMSC population, signal percentage of cell line A: 10% (upper), cell line B: 11% (middle), and cell line C: 17% (lower).

Both groups lacked the expression of hematopoietic markers, namely, CD14, CD34, and CD45, and both groups could express the main MSC markers, namely, CD73, CD90, and CD105 with a strong signal for the three; another 2 markers tested the CD146 which showed a weak signal of 11% on average Figure 5(b) and CD271 which showed a very weak signal of 2% on an average Figure 5(a); this was another reason to choose the CD146 as a confirmatory marker for the gingival connective tissue stem cells, where CD271-positive GMSCs were very rare in the gingival isolated stem cells.

### 3.4. Flow Cytometry Cell Sorting

Using flow cytometry cell sorting module, the cells expressing the CD146 marker were isolated successfully, and the isolated cells were attached to plastic and started to show a fibroblast-like shape the next day. Unfortunately, after 3 days, all the isolated cell dishes showed bacterial contamination, in all the plates; the experiment was repeated three times with the same tragedy; this forced us to resort to magnetic sorting.

### 3.5. Magnetic Cell Sorting

Using a magnetic sorting method for cell isolation, CD146 homogeneous cell population was isolated successfully, cells showed plastic adherence after a longer time than expected, and cells did not show fibroblast-like morphology except after two to three days on average; an explanation might be that the magnetic sorting antibodies hinder the MSC attachment to the plastic; after that, cells behaved normally and showed a colony-forming unit after 12 to 13 days on average; another confirmatory flow cytometry assay was done to ensure the homogeneity of the CD146 cell population, which showed 55% signal (Figures 3(a)–3(c).

### 3.6. Cell Metabolic Activity

This MTT test examined the metabolic activity of the cultured cells and hence its vitality. Both the experiment and the heterogeneous cell groups showed no significant difference between them regarding its metabolic activity (*p* > 0.05; Mann–Whitney *U* test) (Figure 4(c)).

### 3.7. Multilineage Differentiation Potential

GMSCs of both groups cultured in osteogenic induction media for 28 days showed osteogenic differentiation capacity, which was proved by calcification stained by Alizarin Red stain. Cells of both groups cultured in chondrogenic induction media for 28 days showed chondrogenic differentiation capacity proved by cartilage glycoprotein deposits stained by Alcian blue stain. Finally, cells of both groups cultured in adipogenic induction media for 28 days showed lipid deposits proved by lipid droplets stained by Oil Red stain. Control GMSCs cultured in alpha-MEM media with 10% FBS for 28 days did not stain any deposits with three mentioned stains (Figure 4(d)).

### 3.8. Transwell Migration Potential

#### 3.8.1. Migration through Polycarbonate Membrane 8 *μ*m Pore Size

**Figure 6:**
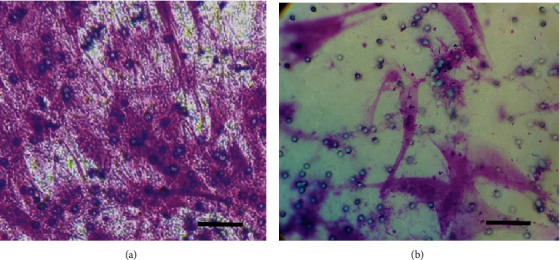
(a) Migrated CD146-positive homogeneous GMSC in the lower compartment of 8 *μ*m perforated polycarbonate membrane toward fetal bovine serum as a chemoattractant; cells stained with crystal violet; 10,000 cells seeded in the upper compartment. (b) Migrated heterogeneous GMSC in the lower compartment of 8 *μ*m perforated polycarbonate membrane toward fetal bovine serum as a chemoattractant; cells stained with crystal violet; 10,000 cells seeded in the upper compartment (40x magnification).

A significantly higher number of CD146-positive homogeneous GMSCs migrated through the membrane compared to the heterogeneous GMSC population toward the 10% FBS chemoattractant, (the *z*-score is 2.50672. The *p* value is 0.01208. The result is significant at *p* < 0.05) (Figures 6(a) and 6(b), Table 1).

#### 3.8.2. Migration through Collagen-Coated PTFE Membrane 0.4 and 3 *μ*m Pore Size

A significantly higher number of CD146-positive homogeneous GMSCs migrated through the 0.4 (the *z*-score is 2.08893. The *p* value is 0.03662. The result is significant at *p* < 0.05) and 3 *μ*m pores (the *z*-score is 2.50672. The *p* value is 0.01208. The result is significant at *p* < 0.05) compared to the heterogeneous GMSCs toward 10% FBS in the lower compartment of the transwell migration chamber as a chemoattractant (Table 1); to be noted, the migration of cells of both groups through 0.4 *μ*m was nonsignificantly less than the migration through 3 *μ*m pores; both were statistically significantly less than migrated cells through the 8 *μ*m pores.

### 3.9. Scanning Electron Microscope

**Figure 7:**
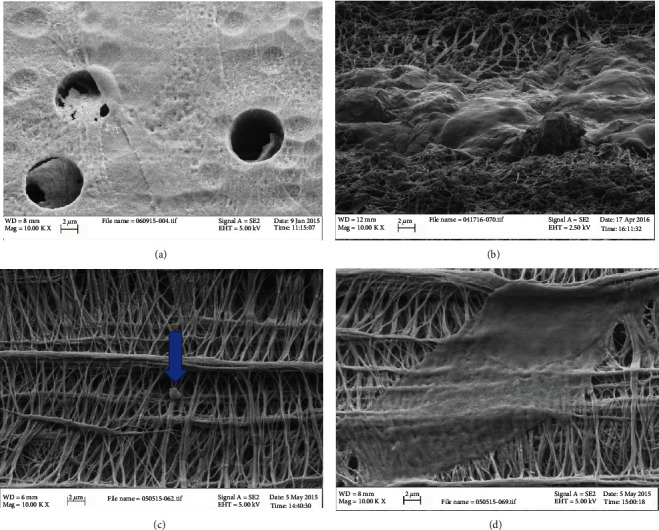
(a) Scanning electron microscope image of migrated GMSCs in the lower compartment of 8-micron pore perforated polycarbonate membrane. (b) Scanning electron microscope image of migrated GMSCs in the lower compartment of 3-micron pore perforated collagen-coated PTFE. (c) Scanning electron microscope image of 0.4-micron pore perforated collagen-coated PTFE showing a GMSC process extending between collagen strands. (d) Scanning electron microscope image of 0.4-micron pore perforated collagen-coated PTFE showing fully migrated GMSC.

No morphological difference was noticed in the cell shape or form or migration pattern through the micropores between the two groups. Both groups' cells looked to be flatter and spread over a larger area over the polycarbonate membrane compared to the collagen membrane. On collagen, the cells of both groups looked more bulbous and extend strands all over the collagen meshwork Figures 7(a)–7(d).

## 4. Discussion

The principle of GTR is to block the migration of the gingival epithelium along the cementum wall of the pocket, creating a space for stabilization of the blood clot to allow the periodontal ligament (PDL) cells to invade the blood clot for the aim of periodontal tissue regeneration [22]. The GTR membrane is hence a physical barrier that has a biologic effect on the healing process of the PDL, affecting the differentiation and proliferation of the mesenchyme, and through clot protection during early stages of healing maintains space for the growing periodontal tissue, to repopulate the wound area with selective tissue populations. Hence, the GTR membrane is considered a biomechanical membrane.

In 2018, Al Bahrawy et al. [4] proved the concept that GMSCs can migrate through microperforated membranes of GTR in the presence of a suitable chemoattractant, while in the absence of the chemoattractant, the membranes were totally occlusive for cell migration; this can be a basis for a new generation of the GTR technique, where a selective barrier membrane was employed, which was occlusive for undesired cells in the gingival tissue, namely, the epithelium and connective tissue cells, and allowed the homing of GMSCs from the gingival tissue to the periodontal wound by utilizing a suitable chemoattractant in the wound area [4].

In the present study, the proliferation and the migration potential of homogeneous CD146-positive GMSC population were compared to a heterogeneous GMSC population of origin; the reason for choosing the CD146 marker to isolate the GMSC population was the unique characteristics of that marker, being not expressed by the fibroblasts and expressed by nearly all the MSC populations, which made it a very good candidate as a marker that insured the stemness of the cell population.

In this study, homogeneous CD146-positive cell population migrated significantly more than the heterogeneous GMSC population, through 0.4 *μ*m, 3 *μ*m, and 8 *μ*m pores of microperforated membranes toward 10% FBS in alpha-MEM media as a chemoattractant; also, the homogeneous population showed nonsignificantly better proliferation capacity than the heterogeneous GMSC population; this could prove the hypothesis that the homogeneous CD146 population can show more proliferation potential and migration chemodynamics through microperforated membranes compared to the heterogeneous GMSC population; this might be explained by a better migration potential of the CD146-positive cells or the existence of a chemoattractant factor in the serum more specific for the CD146-positive cells; this result needs further investigation.

The isolated cells demonstrated all the criteria of the International Society of Cellular Therapy of stem cells, namely, plastic adherence; the ability of colony forming; expression of immunophenotype markers CD105, CD73, and CD90; lack the expression of hematopoietic markers CD45, CD34, and CD14; and finally multipotent differentiation potential [6, 21, 23]. In comparison to the heterogeneous GMSC population, CD146-positive homogeneous GMSCs were similar in every aspect, except for faster proliferation, and a significant number of cells migrated to the lower compartment of the migration chamber during 24-hour period.

In this study, 10% FBS was utilized as the chemoattractant for both the homogeneous and the heterogeneous GMSC populations to assess their migration potential through microperforated membranes. In both groups, cells were seeded in the upper compartment with a concentration of 10,000 cells; this concentration was chosen according to our previous study which proved that adding a greater number of cells in the upper compartment made cell identification and counting absolutely difficult in the lower compartment [4].

GMSCs actively migrated irrespective to the effect of gravitational forces or fluid diffusion forces. In both groups, the rate of cell migration in 24-hour intervals only varied according to the sizes of the pores, where the highest migration rate was through the 8 *μ*m pores and the least through the 0.4 *μ*m pores. GMSCs from both groups did not migrate with statistical significance through the 0.4 *μ*m and 3 *μ*m pores within the same group, but with statistically significant difference between groups in favor of the homogeneous population group; instead, there was a statistically significant difference in the rate of cell migration through the 8 *μ*m compared to 3 *μ*m and 0.4 *μ*m within the same group and with a significant difference between the groups in favor of the homogeneous population group. This peculiar finding suggests that the 8 *μ*m pore size might have a selective migratory effect on GMSCs according to its population homogeneity.

The SEM image analysis was indifferent in morphology between the heterogeneous and the homogeneous groups; both groups showed a fibroblast-like morphology. GMSCs from both groups showed a flatter shape with longer pseudopodia over the polycarbonate membrane, compared to GMSCs migrated through collagen membrane which looked rougher with many extensions to collagen strands; the difference can be explained by the difference in the membrane roughness, the rougher collagen membrane, and the flat polycarbonate membrane [24–26]; this was consistent with previous researches which proved the effect of different substrates on the shape and morphology of the attached cells [27, 28] and even specifically investigated the effect of polycarbonate and collagen substrates on the morphology of the attached cells in Rasmussen et al.'s study [28, 29].

## 5. Conclusion

Homogeneous CD146-positive GMSC populations were more dynamically active in the migration through microperforated membranes and have shorter proliferation time, where 8 *μ*m perforation showed the highest number of migrated cells compared to 0.4 and 3 *μ*m pores. This would throw light on the importance of chemotaxis on homogeneous GMSC migration through the microperforated membrane, using a specific chemoattractant for the homing of specific GMSC population which would migrate more rapidly and proliferate better compared to a nonspecific chemoattractant which would attract less homogeneous or heterogeneous GMSCs to the periodontal wound, a pivotal development in the guided tissue regeneration technique. Studying the effect of different chemotaxis factors on different stem cell lines to choose the best chemoattractive factor is recommended, besides determining the best stem cell line within the GMSC heterogeneous population that can differentiate to multiple cells in the periodontal wound for optimum regeneration results.

## Figures and Tables

**Table 1:** The average number of migrated cells counted in five, random fields at 40x light microscope magnification. For 8 microns, the *z*-score was 2.50672. The *p* value was 0.01208. The result was significant at *p* < 0.05. For 3 microns, the *z*-score was 2.50672. The *p* value was 0.01208. The result was significant at *p* < 0.05. For 0.4 microns, the *z*-score was 2.08893. The *p* value was 0.03662. The result was significant at *p* < 0.05.

Experiment	8 microns		3 microns		0.4 microns	
	CD146+	Heterogeneous	CD146+	Heterogeneous	CD146+	Heterogeneous
1	202^∗^	90	41^∗^	25	3^∗^	2
2	149^∗^	50	40^∗^	22	3^∗^	2
3	229^∗^	45	33^∗^	26	2^∗^	1
4	223^∗^	60	30^∗^	24	3^∗^	2

^∗^A statistically significant difference of GMSC CD146 homogeneous population compared to the heterogeneous GMSC population.

## Data Availability

All the raw data of this study are available for whom is concerned.
